# LncRNA ABHD11-AS1 activates EGFR signaling to promote cervical cancer progression by preventing FUS-mediated degradation of ABHD11 mRNA

**DOI:** 10.1080/15384101.2023.2297591

**Published:** 2023-12-26

**Authors:** Ting Yang, Sijuan Tian, Juan Zhao, Meili Pei, Minyi Zhao, Xiaofeng Yang

**Affiliations:** Department of Obstetrics and Gynecology, The First Affiliated Hospital of Xi’an Jiaotong University, Xi’an City, Shaanxi Province, China

**Keywords:** LncRNA ABHD11-AS1, cervical cancer, ABHD11, EGFR

## Abstract

Cervical cancer is one of the most common gynecological cancers with high metastasis, poor prognosis and conventional chemotherapy. The long non-coding RNA (lncRNA) ABHD11 antisense RNA 1 (ABHD11-AS1) plays a vital role in tumorigenesis and is involved in cell proliferation, differentiation, and apoptosis. Especially for cervical cancer, the functions and mechanisms of ABHD11-AS1 are still undetermined. In this study, we explored the role and underlying mechanism of ABHD11-AS1 in cervical cancer. We found that ABHD11-AS1 is highly expressed in cervical cancer tissue. The roles of ABHD11-AS1 and EGFR have investigated the loss of function analysis and cell movability in SiHa and Hela cells. Knockdown of ABHD11-AS1 and EGFR significantly inhibited the proliferation, migration, and invasion and promoted apoptosis of SiHa and Hela cells by up-regulating p21 and Bax and down-regulating cyclin D1, Bcl2, MMP9, and Vimentin. ABHD11-AS1 knockdown could decrease the expression of EGFR. In addition, ABHD11-AS1 could regulate the EGFR signaling pathway, including p-EGFR, p-AKT, and p-ERK. Spearman’s correlation analysis and cell experiments demonstrated that ABHD11 was highly expressed in tumor tissue and partially offset the effect of shABHD11-AS1 on the proliferation, migration, and invasion of SiHa and Hela cells. Then, RNA pulldown was used to ascertain the mechanisms of ABHD11-AS1 and FUS. ABHD11-AS1 inhibited ABHD11 mRNA degradation by bounding to FUS. A subcutaneous xenograft of SiHa cells was established to investigate the effect of ABHD11-AS1 in tumor tissue. Knockdown of ABDH11-AS1 inhibited tumor growth and decreased the tumor volume. ABHD11-AS1 knockdown inhibited the expression of Ki67 and Vimentin and up-regulated the expression of Tunel. Our data indicated that ABHD11-AS1 promoted cervical cancer progression by activating EGFR signaling, preventing FUS-mediated degradation of ABHD11 mRNA. Our findings provide novel insights into the potential role of lncRNA in cervical cancer therapy.

## Introduction

Cervical cancer is one of the most common gynecological cancers and is blamed for 311,000 deaths worldwide annually [1,2]. It is the second leading cause of death among women [3,4]. Cervical cancer is usually caused by various risk factors, such as sexually transmitted infections, socioeconomic factors, and family history [5]. However, the most apparent cause is the infection with human papillomavirus (HPV) [6,7]. The current primary treatment for cervical cancer contains radiotherapy, surgical resection, and locally targeted therapy [8,9]. These therapies are only effective in cases of early-stage cervical cancer. However, they have limited efficacy in the middle-advanced stage or metastatic cervical cancer because of their drug resistance, metastases, and so on [10,11]. Consequently, there is an urgent demand to seek novel biomarkers for better diagnosis and treatment of cervical cancer.

Long noncoding RNAs (lncRNAs) have received widespread attention as new biomarkers. LncRNAs are RNA transcripts > 200 nucleotides that do not encode protein [12,13]. LncRNAs play crucial roles in cellular processes, including cell cycle, differentiation, proliferation, and metabolism [14–16]. LncRNAs can modulate transcription and translation by interacting with DNA, RNA, or protein [17–19]. For instance, lncRNA PTENP1 suppresses cervical cancer progression by promoting PTEN expression, inhibiting cell proliferation, and inducing cell apoptosis [20]. LncRNA SNHG1 is highly expressed in cervical cancer and could encourage the development of cervical cancer cells [21]. In addition, lncRNA LAMTOR5-AS1 decreases expression levels in cervical cancer. Overexpression of LAMTOR5-AS1 effectively suppresses the growth of cervical cancer cells by sponging miR-210-3p [22]. Hence, the specific mechanism of lncRNAs in cervical cancer occurrence and progression requires further investigation.

LncRNA ABHD11-AS1, located at human chromosome 7 q11.23, has been proven to be related to many cancers, including cervical cancer, endometrial carcinoma, bladder cancer, and colorectal cancer [23,24]. It has been reported that ABHD11-AS1 promoted papillary thyroid carcinoma tumor progression by regulating the EPS15L1/EGFR pathway [25]. In addition, ABHD11-AS1 promoted cell proliferation and increased tumorigenicity in endometrial carcinoma by targeting cyclinD1 [26]. ABHD11-AS1 is over-expressed in colorectal cancer and advanced tumor progression by targeting ITGA5/PI3K/Akt signaling pathway [27].

In this study, we examined the expression of ABHD11-AS1 in different cervical cancer cells. The biological functions and molecular pathways of ABHD11-AS1 were investigated in cervical cancer cells. Our results suggested that ABHD11-AS1 played an important role in cervical cancer progression by targeting EGFR and could be a novel potential target or biomarker for diagnosing and treating cervical cancer.

## Material and methods

### Cell lines and culture conditions

Human normal cervical epithelial H8 cells and cervical cancer cells (SiHa, C-33A, Ca Ski, and Hela) were obtained from the Chinese Academy of Sciences (Beijing, China). Dulbecco's modified Eagle’s medium supplemented with 10% fetal bovine serum was used to culture cells at 37°C in 5% CO_2_.

### Cell growth and proliferation assays

The cell viability was assessed using a Cell Counting Kit 8 (CCK8), following the kit’s instructions. Optical density at 450 nm absorbance of each well was detected by microplate spectrophotometer.

Cell colonies were fixed with 4% paraformaldehyde for 1 hour for the colony formation assay. Then, colonies were stained with crystal violet. The territories of each well were counted.

### In situ hybridization (ISH)

ISH was used to evaluate ABHD11-AS1 expression levels in paracancerous and cervical carcinoma tissue. The tissue was fixed in 10% paraformaldehyde at room temperature for 24 h. Then, paraffin blocks were cut into 5 μm sections. Sections were deparaffinized with xylene and graded ethanol solutions. Sections were incubated with 3% H_2_O_2_ for 10 minutes at room temperature to block endogenous peroxidase and washed 3 times with ddH_2_O. Slides were incubated with 3% citric acid and freshly diluted pepsin for about 10 minutes at room temperature, then washed 3 times with PBS and ddH_2_O. Sections were pre-hybridized for 2 h in a box with 20 ml of 20% glycerin. Then, sections were hybridized with 20 μl hybridization liquid, washed once with 2×SSC, 0.5×SSC, and 0.2×SSC and blocked for 30 min at 37°C. Sections were incubated with biotin-digoxigenin for 2 h at room temperature. After being washed 3 times with PBS, sections were incubated with Strept Avidin-Biotin Complex (SABC) for 20 minutes at 37°C. Then, sections were subjected to DAB, followed by hematoxylin redye and vitrification with dimethylbenzene. The results were obtained using the EnVision Detection System.

### RNA extraction and real-time PCR

Total RNA was extracted from tumors and cells using TRIzol reagent (Invitrogen, USA). The total was reverse transcribed into cDNA using a PrimeScript RT reagent kit (Tiangen, China). The mRNA expression was determined using the SYBR@ Green reagent (Roche, Indianapolis, IN, USA) and normalized using GAPDH as an internal control.

### Apoptosis assay

Cell apoptosis assay was performed following the instructions of FITC Annexin V Apoptosis Detection Kit I (BD Pharmingen). The results were obtained on the FACS Canto II flow cytometer (BD Biosciences).

### Transwell migration and invasion assay

The Transwell chamber system, purchased from Corning (USA), was used to detect cell migration and invasion. For migration, cells were added into the upper chamber containing 200 μl FBS-free medium. Then 600 μl media with 20% FBS were added outside the chamber. For invasion, the upper chamber was pre-coated with Matrigel. Then, cells were seeded into the upper chamber. After incubation for 48 hours, cells were fixed with methanol and then stained with hematoxylin. Cell numbers were imaged and counted by microscope (Leica DM 2500).

### Western blot analysis

The protein sample was isolated from the tumor and cells with RIPA buffer (Beyotime, China). BCA kit (Thermo Fisher Scientific, USA) measured protein concentration. An equal amount of denatured protein sample was separated onto 10–12.5% SDS-PAGE gel and subsequently electro-transferred to the FVDF membrane (EDM Millipore, Billerica, MA). The membranes were blocked with 5% nonfat dry milk in TBST at room temperature for 1 hour. Then, the membranes were incubated with primary antibodies at 4°C overnight and subsequently incubated with secondary antibodies at 37°C for 1 h. Protein blots were visualized using Chemilumines-cent Substrate. Image software was used for protein semi-quantitative analysis. Densitometric values were normalized to GAPDH.

### Immunohistochemistry (IHC)

IHC was performed to detect the expression of EGFR, ABHD11, FUS, Ki-67, Tunel, and Vimentin in paracancerous and cervical carcinoma tissue. At room temperature, the tissue was fixed in 10% paraformaldehyde for 24 h. After dehydrating in graded ethanol solutions, the tissue cleared with xylene and embedded into paraffin wax. Finally, paraffin blocks were cut into 5 μm sections and washed with PBS 3 times for subsequent experiments. Then, slides were incubated overnight at room temperature with primary antibodies. Then, slides were incubated for 20 min with a secondary antibody. Finally, The slides were colored with DAB Peroxidase Substrate. Slides were analyzed and imaged on a brightfield microscope.

### RNA immunoprecipitation (RIP)

Briefly, cells were collected for nuclear isolation. First, cells were incubated with the primary or IgG antibody for 4 h at 4°C in a buffer with RNase inhibitors. Then, cells were incubated with protein A/G magnetic beads overnight at 4°C. Compounds were incubated with proteinase K digestion to remove protein. Finally, RNA was extracted using phenol-chloroform and analyzed by qRT-PCR.

### RNA pull-down assay

The RNA pull-down assay used a RNA-Protein Pull-Down Kit (Thermo Scientific, cat# 20164). Briefly, RNA probes were labeled by biotinylated cytidine bisphosphate and T4 RNA ligase (Thermo Scientific, cat# 20163). Then, labeled RNA was incubated with agitation for 30 minutes at room temperature to bind to streptavidin magnetic beads. Protein lysates were incubated with RNA-bound beads for 1 h at 4°C, then washed with wash buffer. The RNA-protein collections were used for western blotting.

### Animal models

The Ethics Committee of The First Affiliated Hospital of Xi’an Jiaotong University approved this animal study and all animal experiments. Animal experiments were performed by the National Institutes of Health Guide for the Care and Use of Laboratory Animals. For subcutaneous tumorigenesis experiments, BALB/C nude mice around 4 weeks were used to establish an *in vivo* model by subcutaneously injecting SiHa cells. The mice in the shNC + Gefitinib group were orally administered Gefitinib. Xenografts were measured weekly. Five weeks later, all mice were sacrificed, and the weight of each tumor was measured. Tumor tissues were used for the following experiment. For metastasis experiments, 4-week-old BALB/C nude mice were injected via the tail vein with SiHa cells. After six weeks, nude mice were sacrificed by cervical dislocation. Then, lung tissues were stained with H&E for histological examination.

### Statistical analysis

Every experiment has been performed at least triplicates repeated. Means ± standard deviation (SD) was expressed as the value. The data in this study were processed by SPSS statistical software using ANOVA with Bonferroni correction for differences in multiple groups and Student’s t-test for the difference of mean values between two groups. The value of *p* < 0.05 was considered a statistically significant difference.

## Result

### EGFR and ABHD11-AS1 are highly expressed in cervical carcinoma tissue and cell

**Figure 1.**
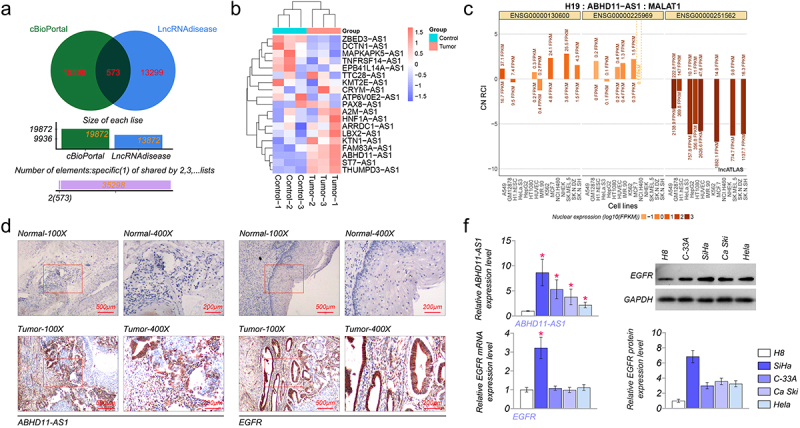
EGFR and ABHD11-AS1 are highly expressed in cervical cancer. (a) The venn diagram of common lncRnas between cervical cancer and EGFR-related. (b) The heat map of expression of 19 differentially expressed lncRnas between normal and tumor tissue. (c) LncATLAS analyzed the subcellular localization of ABHD11-AS1. (d) The subcellular localization and expression of ABHD11-AS1 and EGFR by ISH and IHC, respectively. (E) ABHD11-AS1 and EGFR expression levels in H8, SiHa, C-33A, Ca Ski, and Hela cell lines via qRT-PCR or western blot. *p < 0.05 versus H8.

We obtained 19,299 EGFR-related antisense lncRNAs from the cBioPortal database. Meanwhile, antisense lncRNAs were analyzed in cervical cancer from the LncRNAdisease v2.0 database, and 13,299 candidate lncRNAs were highly expressed in cervical cancer. Next, Venn analysis was performed to obtain 573 overlapping antisense lncRNAs in the two databases (Figure 1(a)). Finally, 19 antisense lncRNAs were obtained by the screening criteria of Spearman’s Correlation (absolute value) >0.3, p-Value <10^−6^, and q-Value <10^−5^. Subsequently, qRT-PCR was used to examine the expression of these lncRNAs between paracancerous tissue and cervical carcinoma tissue. As shown in (Figure 1(b)), the expression of ABHD11-AS1 has the most significant difference between paracancerous tissue and cervical carcinoma tissue. Therefore, ABHD11-AS1 was selected for further investigation. The clinicopathologic features of the 20 patients are shown in Table 1. The subjects were divided into an expression group (*n* = 10) and a high expression group (*n* = 10) according to the expressing median value in cervical carcinoma tissues. The correlation of ABHD11-AS1 expression with clinicopathological data was assessed and summarized in Table 1. No significant association between age (*p* = 0.635), menopause (*p* = 0.635), histology (*p* = 0.815), differentiation (*p* = 0.178), lymph node (*p* = 0.185), and ABHD11-AS1 expression level was observed; however, significant correlations were observed between ABHD11-AS1 expression and grade number (*p* = 0.041), tumor size (*p* = 0.025).

**Table 1.** The clinicopathologic features of the 20 patients.

Chinicopathological characteristics	Total	ABHD11-AS1 Low expression	ABHD11-AS1 High expression	P value
Age				0.635
<50	11	6	5	
≥50	9	4	5	
Menopause				0.635
Yes	11	5	6	
No	9	5	4	
Histology				0.815
Squamous cell cancer	13	7	6	
Adenocarcinoma	4	2	2	
Others	3	1	2	
Differentiation				0.178
Well	9	6	3	
Poor	11	4	7	
Grade number				0.041
I	6	5	1	
II	7	4	3	
III	7	1	6	
Tumor size				0.025
<4 cm	11	8	3	
≥4 cm	9	2	7	
Lymph node				0.185
Negative	11	7	4	
Positive	9	3	6	

The subcellular localization of ABHD11-AS1 was analyzed by the lncATLAS website (http://lncatlas.crg.eu) [28]. The result demonstrated that ABHD11-AS1 was distributed in the cytoplasm and nucleus (Figure 1(c)). Subsequently, the result was further validated utilizing ISH and IHC. We found that ABHD11-AS1 was mainly located in the cytoplasm but a minor in the nucleus via ISH. EGFR was situated on the cytomembrane and cytoplasm by IHC. Also, the results of ISH showed that the expression of ABHD11-AS1 was up-regulated in cervical carcinoma tissue, compared with paracancerous tissue (Figure 1(d)). The expression of EGFR was up-regulated in cervical carcinoma tissue by IHC (Figure 1(d)).

Meanwhile, to further confirm this finding, the expression level of ABHD11-AS1 and EGFR were determined in cervical cancer cell lines (SiHa, C-33A, Ca Ski, and Hela) and normal cervical epithelial H8 cell lines. Compared to the expression level in H8, a higher mRNA level of ABHD11-AS1 was observed in the cervical cancer cell lines. Among these four cervical cancer cell lines, SiHa had the highest ABHD11-AS1 expression, and Hela had the lowest ABHD11-AS1 expression (Figure 1(e)). Then, the results of qRT-PCR and western blot suggested that the expression of EGFR was highest in SiHa cells (Figure 1(e)). Thus, SiHa and Hela were selected for further experiments.

### Knockdown of EGFR and ABHD11-AS1 inhibit the movability of cervical cancer cells

**Figure 2.**
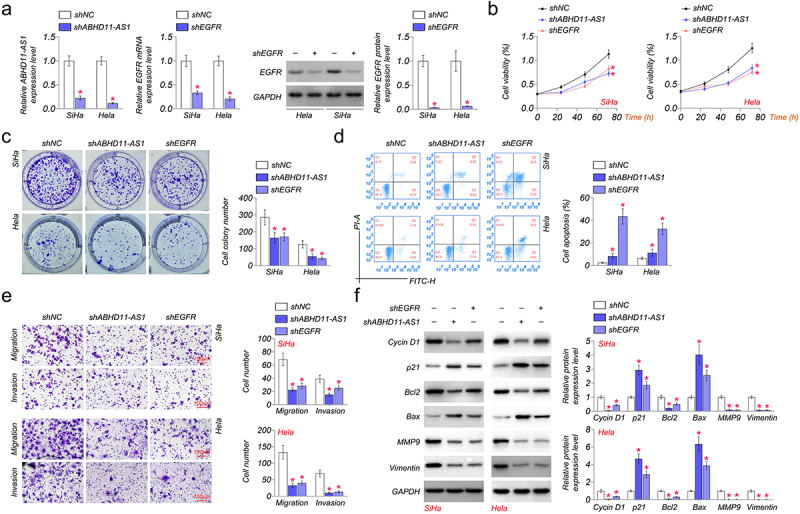
EGFR and ABHD11-AS1 promote cervical cancer cells’ proliferation, migration, and invasion capacities. (a) qRT-PCR or western blot revealed ABHD11-AS1 and EGFR expression levels in SiHa and hela cells with shABHD11-AS1 and shEGFR, respectively. (b-c) The viability of SiHa and hela cells was examined by CCK8 (b) and colony formation assays (C). (d) The flow cytometry analysis results and apoptosis rates of SiHa and hela cells with shABHD11-AS1 and shEGFR. (e) Transwell migration and transwell invasion assays were performed to detect the effects of shABHD11-AS1 and shEGFR on cell migration and invasion, respectively. (f) Cycin D1, p21, Bcl2, Bax, MMP9, and Vimentin were expressed in SiHa and hela cells with shABHD11-AS1 and shEGFR. *p < 0.05 versus shNC.

To investigate the biological functions of ABHD11-AS1 in cervical cancer, shRNAs were utilized to inhibit the expression of ABHD11-AS1 and EGFR in SiHa and Hela, and the silencing efficiency was confirmed by qRT-PCR or western blot. Compared with the expression of ABHD11-AS1 and EGFR in shNC cells, the lower ABHD11-AS1 or EGFR expression level was observed in shABHD11-AS1 or shEGFR transfected SiHa and Hela cells (Figure 2(a)).

We detected the proliferation, apoptosis, migration, and invasion of SiHa and Hela cells transfected with shNC, shABHD11-AS1, or shEGFR. Our results indicated that shABHD11-AS1 or shEGFR notably suppressed the proliferation of SiHa and Hela cells compared to shNC by CCK8 and colony formation assays (Figure 2(b,c)). Knocked down ABHD11-AS1 or EGFR expression significantly promoted apoptosis of SiHa and Hela cells by flow cytometry (Figure 2(d)). The transwell assays were performed to detect the migration of SiHa and Hela cells. We found that the migration ability and number were also significantly inhibited by shABHD11-AS1 or shEGFR in SiHa and Hela (Figure 2(e)). Cell invasion was examined by using a transwell invasion assay. The results demonstrated that shABHD11-AS1 or shEGFR significantly attenuated the invasion ability of SiHa and Hela cells (Figure 2(e)).

Furthermore, we detected the expression of proliferation markers (cyclin D1 and p21), apoptosis-related protein (Bcl2 and Bax), and migration-related protein (MMP9 and Vimentin) by western blot. The results showed that shABHD11-AS1 or shEGFR induced up-regulation of p21 and Bax in SiHa and Hela cells, while shABHD11-AS1 or shEGFR down-regulated the expression of cyclin D1, Bcl2, MMP9, and Vimentin (Figure 2(f)). The results above indicated that the knockdown of EGFR and ABHD11-AS1 inhibits cervical cancer cell proliferation, migration, and invasion capacities.

### ABHD11-AS1 activates the EGFR signaling pathway

**Figure 3.**
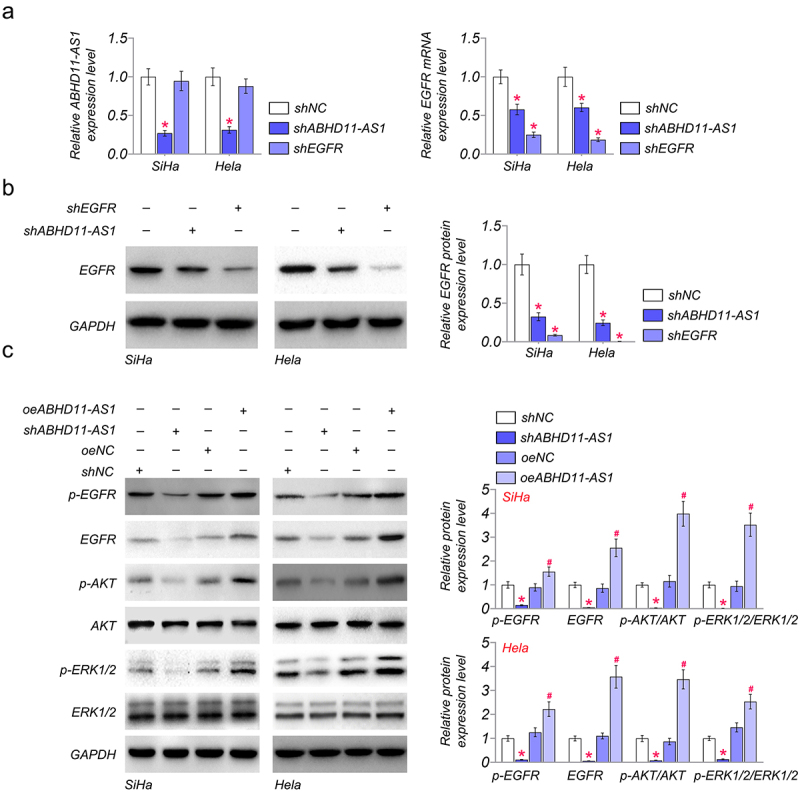
ABHD11-AS1 activates the EGFR signaling pathway. (a) the mRNA levels of ABHD11-AS1 and EGFR were examined via qRT-PCR in SiHa and hela cells with shABHD11-AS1 and shEGFR. (b) The protein levels of EGFR were examined by western blot in SiHa and hela cells with shABHD11-AS1 and shEGFR. (c) Western blot assay was performed to detect the expression of p-EGFR, EGFR, p-AKT, AKT, ERK1/2, and p-ERK1/2 in SiHa and hela cells with shABHD11-AS1 and oeABHD11-AS1. *p < 0.05 versus shNC; ^#^*p* < 0.05 versus oeNC.

To investigate the relation between ABHD11-AS1 and EGFR, we examined the expression of ABHD11-AS1 and EGFR in SiHa and Hela cells. The results of qRT-PCR showed that the expression of ABHD11-AS1 was not significantly affected by shEGFR in SiHa and Hela cells, compared with shNC. However, shABHD11-AS1 could down-regulated the mRNA level of EGFR by qRT-PCR (Figure 3(a)). Subsequently, we further detected the protein expression of EGFR in SiHa and Hela cells by western blot. The results showed that shABHD11-AS1 significantly inhibited the protein expression of EGFR (Figure 3(b)).

To further investigate the regulation of ABHD11-AS1 on the EGFR signaling pathway, ABHD11-AS1 was overexpressed or knocked down in SiHa and Hela cells. The western blot results showed that shABHD11-AS1 reduced phosphorylation of EGFR and its downstream signaling components, such as p-AKT and p-ERK1/2, in SiHa and Hela cells (Figure 3(c)). However, oeABHD11-AS1 could promote the phosphorylation of EGFR, AKT, and ERK1/2 (Figure 3(c)), which indicates that ABHD11-AS1 activates the EGFR signaling pathway via phosphorylation of EGFR.

### ABHD11-AS1 positively regulates ABHD11 to promote cervical cancer

**Figure 4.**
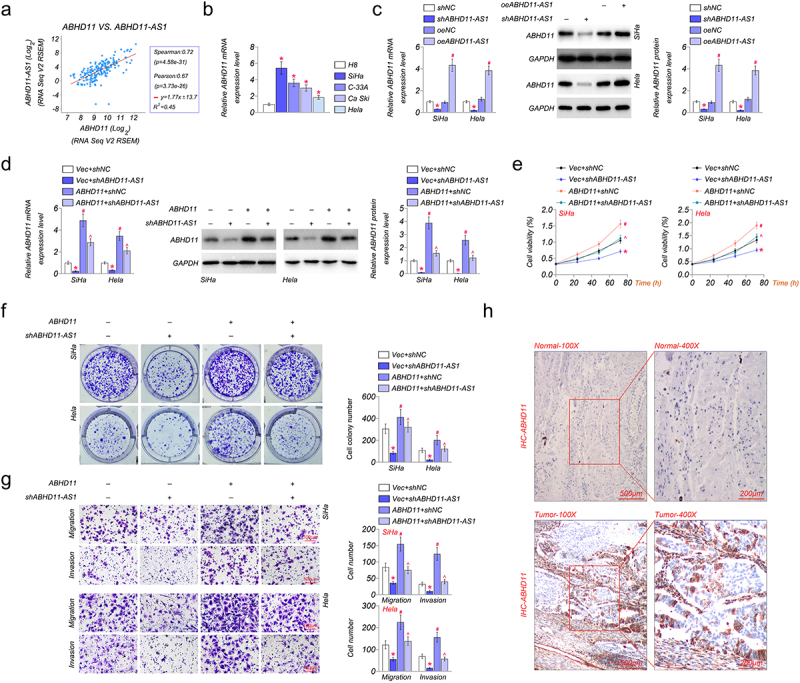
ABHD11-AS1 positively regulates ABHD11 to promote cervical cancer. (a) Spearman’s correlation analysis revealed that the expression levels of ABHD11 and ABHD11-AS1 exhibited a positive correlation (*p* < 0.001). (b) The mRNA levels of ABHD11 in H8, SiHa, C-33A, Ca Ski, and hela cells by qRT-PCR. *p < 0.05 versus H8. (c) qRT-PCR and western blot revealed the mRNA and protein levels of ABHD11 in SiHa and hela cells with shABHD11-AS1 and oeABHD11-AS1. *p < 0.05 versus shNC; ^#^*p* < 0.05 versus oeNC. (d) The effects of ABHD11 and shABHD11-AS1 on the mRNA expression levels of ABHD11 were measured by qRT-PCR and western blot. (e-f) the viability of SiHa and hela cells was examined by CCK8 (e) and colony formation assays (f). (g) The migration and invasion of SiHa and hela cells were detected by transwell. (h) The expression of ABHD11 in cervical carcinoma tissue and paracancerous tissue by IHC. *p < 0.05 versus Vec+shNC; ^#^*p* < 0.05 versus Vec+shABHD11-AS1; ^*p* < 0.05 versus ABHD11+shNC.

The regulation of ABHD11-AS1 was predicted in the UCSC Genome Browser (http://genome.ucsc.edu/). The result showed that ABHD11-AS1 might regulate the expression of a nearby gene, ABHD11. Moreover, Spearman’s correlation analysis revealed a positive correlation between the expression levels of ABHD11-AS1 and ABHD11 in cervical cancer (Figure 4(a)). In addition, there was a higher expression level of ABHD11 in cervical cancer cells compared with that in normal cells. Among these cervical cancer cells, SiHa had the highest ABHD11 expression, and Hela had the lowest ABHD11 expression (Figure 4(b)). Subsequently, the expression of ABHD11 was detected in SiHa and Hela cells by qRT-PCR and western blot. The mRNA and protein levels of ABHD11 were down-regulated in SiHa and Hela cells treated with shABHD11-AS1. And oeABHD11-AS1 could up-regulate the mRNA and protein levels of ABHD11 (Figure 4(c)). However, ABHA11 partially offset the effect of shABHD11-AS1 on both the mRNA and protein of ABHD11 (Figure 4(d)).

The effect of ABHD11-AS1 and ABHD11 on the capacity of cell proliferation, migration, and invasion was investigated in SiHa and Hela cells by CCK8 and colony formation assays. The results suggested that shABHD11-AS1 inhibited cell proliferation of SiHa and Hela cells, and ABHD11 could promote cell proliferation (Figure 4(e,f)). The migration and invasion of SiHa and Hela cells were detected by transwell invasion assays. The results showed that shABHD11-AS1 significantly inhibited migration and invasion of SiHa and Hela cells, and ABHD11 partially offset the effect of shABHD11-AS1 and promoted migration and invasion (Figure 4(g)). The expression of ABHD11 was up-regulated in cervical carcinoma tissue by IHC, compared with paracancerous tissue (Figure 4(h)).

### ABHD11-AS1 inhibits ABHD11 mRNA degradation by binding to FUS

**Figure 5.**
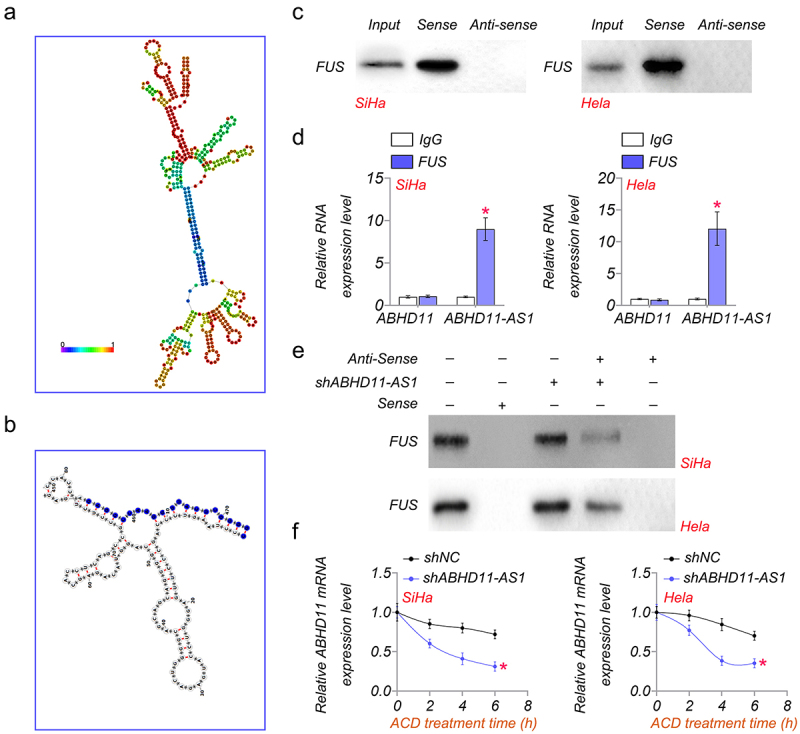
ABHD11-AS1 inhibits ABHD11 mRNA degradation by binding to FUS. (a) the secondary structure diagram of ABHD11-AS1. (b) The binding site of FUS in ABHD11-AS1. (c) RNA pulldown analysis of the interaction of ABHD11-AS1 and FUS. (d) RIP analysis of the interaction between FUS and ABHD11-AS1. (e) RNA pulldown assay was performed to determine the interaction of FUS and ABHD11-AS1 in SiHA and hela cells. (f) The relative mRNA level of ABHD11 in SiHa and hela cells was treated with actinomycin D (2.5 μM) at indicated time points. *p < 0.05 versus shNC.

The secondary structure diagram of ABHD11-AS1 is shown in (Figure 5(a)). We found that there was a FUS binding site in ABHD11-AS1. As shown in (Figure 5(b)), the binding site was from the amino acid at position 454 to position 474, according to the prediction of AnnoLnc2 (http://annolnc.gao-lab.org/) [29]. In addition, RNA pulldown and RIP analysis also showed that ABHD11-AS1 could directly bind to FUS in SiHa and Hela cells (Figure 5(c,d)). To verify this result, RNA pulldown was performed in control or shABHD11-AS1 cells. The results suggested that shABHD11-AS1 significantly promoted ABHD11-AS1 binding to FUS (Figure 5(e)). In addition, shABHD11-AS1 resulted in accelerated ABDH11-AS1 degradation as examined by mRNA levels after being treated with actinomycin D (ACD) (Figure 5(f)). These results indicated that ABHD11-AS1 is bound to FUS to inhibit ABHD11 mRNA degradation.

### The inhibition of ABHD11-AS1 suppresses tumor growth

**Figure 6.**
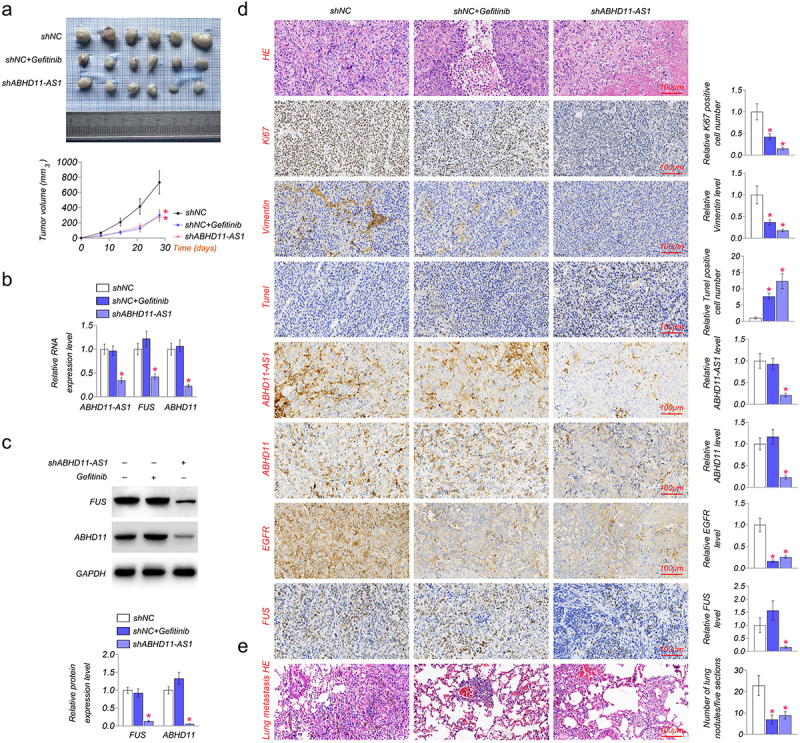
The inhibition of ABHD11-AS1 suppresses tumor growth. (a) the xenografts of SiHa cells were collected in the fifth week. shABHD11-AS1 significantly suppressed the tumor volume as compared to the shNC group. (b) qRT-PCR results revealed ABHD11-AS1, FUS and ABHD11 mRNA expression in tumor tissue by shABHD11-AS1. (c) Western blot was performed to examine the protein levels of FUS and ABHD11 in tumor tissue by shABHD11-AS1. (d) IHC of ki-67, Vimentin, Tunel, ABHD11, EGFR, FUS expression, ISH of ABHD11-AS1 in tumor tissue, HE staining of tumor tissue. (e) The metastasis in lung tissue of nude mice constructed with tail vein injection by HE staining. *p < 0.05 versus shNC.

The role of ABHD11-AS1 was also investigated in nude mice *in vivo*. As shown in (Figure 6(a)), shABDH11-AS1 and EGFR inhibitor, gefitinib significantly inhibited xenograft growth and decreased xenograft volume as compared to the shNC group. Then, we examined the expression of ABHD11-AS1, FUS, EGFR, and ABDH11 in xenograft *in vivo*. The result showed that shABHD11-AS1 markedly down-regulated the expression level of ABHD11-AS1, FUS, ABHD11, and EGFR, but gefitinib did not affect the expression level of ABHD11-AS1, FUS and ABHD11 (Figure 6(b–d)). We examined the expression of proliferation, apoptosis, and migration-related proteins in SiHa cell tumors by IHC based on the above results. The results suggested that gefitinib and shABHD11-AS1 could inhibit the expression of Ki-67 and Vimentin and up-regulated the expression of Tunel (Figure 6(d)). In addition, we observed the effect of ABHD11-AS1 on metastatic ability in lung tissue constructed with tail vein injection. HE staining showed that shABHD11-AS1 significantly decreased the number of lung metastases (Figure 6(e)). These results indicated that lncRNA ABHD11-AS1 promotes cervical cancer via FUS/ABHD11/EGFR axis.

## Discussion

Cervical cancer is the most common malignancy affecting women and seriously endangers women’s health [30,31]. Therefore, it is crucial to seek novel biomarkers or targets for better diagnosis and treatment of cervical cancer. LncRNAs are a class of noncoding RNAs and have important roles in regulating biological processes [31]. However, the mechanism underlying lncRNA in cervical cancer remains elusive. This study investigated the natural function and underlying regulatory agencies of lncRNA ABHD11-AS1 in cervical cancer.

The epidermal growth factor receptor (EGFR), a growth-factor-receptor tyrosine kinase, belongs to the ErbB family of tyrosine kinase receptors [32,33]. EGFR is a critical protein in cell proliferation, differentiation, and cancer development. The over-expression of EGFR has been proven to promote the expansion of tumor cells and inhibit apoptosis, leading to tumor progression [34,35]. EGFR inhibitor has been widely used in the clinical field of cervical cancer [36]. We obtained 19 differentially expressed lncRNAs associated with EGFR and cervical cancer. In these lncRNAs, ABHD11-AS1 expression level was the highest in cervical carcinoma, compared with paracancerous tissue. ABHD11-AS1 is essential in cancer progression, diagnosis, and treatment. Zhu et al. reported that ABHD11-AS1 in the serum of cervical carcinoma patients was higher than that of healthy people [37]. So, we selected ABHD11-AS1 for further investigation. Our results were consistent with the above report. We found that ABHD11-AS1 expression increased in cervical carcinoma tissue, suggesting that ABHD11-AS1 could act as a potential diagnostic or prognostic biomarker for cervical cancer. Then, ABHD11-AS1 expression was detected in different cervical cancer cell lines. SiHa and Hela cell lines have the highest and lowest ABHD11-AS1 expressions, respectively.

We knocked down the expression of ABHD11-AS1 and EGFR to study the biological function of ABHD11-AS1. Hou et al. reported that ABHD11-AS1 expression was abnormally high in cervical cancer cells. The vivo experiments proved that ABHD11-AS1 knockdown impeded tumor growth [38]. Our results suggested that shABHD11-AS1 and shEGFR could significantly inhibit the viability, proliferation, migration, and invasion and induce cell apoptosis of SiHa and Hela cells. These results indicated that down-regulation of ABHD11-AS1 could suppress tumor progression. In addition, we examined the expression of proliferation, apoptosis, and migration-related protein in SiHa and Hela cells. ABHD11-AS1 had similar effects with EGFR. shABHD11-AS1 or shEGFR up-regulated the expression of p21 and Bax and inhibited Cyclin D1, Bcl2, MMP9, and Vimentin in SiHa and Hela cells.

We further investigated the effect of ABHD11-AS1 on the regulation of EGFR signaling. Dysregulation of the EGFR leads to cell proliferation, invasion, and metastasis in tumor tissue [39–41]. Several lncRNAs participate in the regulation of the EGFR signaling pathway. It has been reported that lncRNA KRT16P2 regulated laryngeal squamous cell carcinoma cell proliferation, invasion, and migration by regulating lncRNA KRT16P2/miR-1294/EGFR axis [42]. In addition, lncRNA CASC9 promoted the progression and development of gastric cancer by regulating the miR-370/EGFR axis [43]. EGFR signaling includes AKT and ERK molecules. ERK is a signal transducer growth factor widely involved in biological responses. It has been reported that ERK1/2 is aberrantly expressed and activated in cervical cancer [44,45]. AKT is a serine/threonine kinase that mediates many biological functions, including cell proliferation and apoptosis. Up-regulation of AKT activity has been reported in cervical cancer [46]. In our study, shABHD11-AS1 could down-regulated the expression of EGFR. However, knockdown EGFR has no significant effect on the expression of ABDH11-AS1. Subsequently, we found that knockdown ABHD11-AS1 suppressed the phosphorylation of EGFR, AKT, and ERK1/2. And over-expression ABHD11-AS1 has the opposite function. Hence, we demonstrated that ABHD11-AS1 activates EGFR signaling to promote tumorigenesis.

ABHD protein family members play critical roles in cancers. ABHD11 is a potential biomarker of various malignant tumors, such as lung adenocarcinoma and breast cancer [47–49]. We predicted ABHD11-AS1 might regulate ABHD11, which is near ABHD11-AS1. ABHD11 was highly expressed in cervical cancer cells. Knockdown ABHD11-AS1 could down-regulated the expression of ABHD11. We also found that ABHD11 promoted cell proliferation, migration, and invasion of cervical cancer cells. ABHD11 could partially offset the effect of knockdown ABHD11-AS1.

ABHD11-AS1 has a FUS binding site by prediction of AnnoLnc2. FUS, an RNA-binding protein, is a member of the FET protein family. Previous studies have confirmed that it plays a crucial role in various biological behaviors, including DNA recombination and repair and pre-mRNA splicing [50,51]. Moreover, FUS has been found to participate in the development of several cancers. For instance, by interacting with NEAT1, the low expression of FUS/TLS induces cell apoptosis and inhibits cell growth in breast cancer [52]. FUS/TLS participates in the mediation of cell-cycle progression androgen-dependent and cancer growth in prostate cancer [53]. CircRNA_0000285 could enhance the proliferation and metastasis of cervical cancer by up-regulating FUS [54]. In this study, the results of RNA pulldown showed that ABHD11-AS1 could directly bind to FUS to inhibit ABHD11 mRNA degradation.

To determine the effect of ABHD11-AS1 on tumorigenesis in vivo, we constructed mouse-transplanted tumor models using nude mice transplanted with SiHa cells. Knockdown of ABHD11-AS1 dramatically suppressed tumor growth. Gefitinib, an EGFR inhibitor, also obviously decreased tumor volume. Gefitinib does not affect the expression of ABHD11-AS1, FUS, and ABHD11. However, the knockdown of ABHD11-AS1 could down-regulated the expression of FUS and ABHD11. These results suggested that ABHD11-AS1 binding to FUS inhibits ABHD11 mRNA degradation to activate EGFR signaling.

In conclusion, ABHD11-AS1 knockdown suppressed the growth and invasion capability of cervical cancer cells by promoting FUS-mediated degradation of ABHD11 mRNA to activate EGFR signaling. These findings have revealed the mechanisms of ABHD11-AS1, which may act as a possible biomarker or target for diagnosing and treating cervical cancer.

## Data Availability

The authors declare that all data supporting the findings of this study are available within the paper, and any raw data can be obtained from the corresponding author upon request.
